# Are Non-Pharmacological Interventions Effective in Reducing Drug Use and Criminality? A Systematic and Meta-Analytical Review with an Economic Appraisal of These Interventions

**DOI:** 10.3390/ijerph13100966

**Published:** 2016-09-29

**Authors:** Amanda E. Perry, Rebecca Woodhouse, Matthew Neilson, Marrissa Martyn St James, Julie Glanville, Catherine Hewitt, Dominic Trépel

**Affiliations:** 1 Department of Health Sciences, Mental Health and Addictions Research Group, ARRC Building, 2nd Floor, University of York, Heslington, York YO10 5DD, UK; Rebecca.woodhouse@york.ac.uk (R.W.); mjneilson@gmail.com (M.N.); dominic.trepel@york.ac.uk (D.T.); 2 Health Economics and Decision Science, School for Health and Related Research (ScHARR), University of Sheffield, Regent Court, 30 Regent Street, Sheffield S1 4DA, UK; m.martyn-stjames@sheffield.ac.uk; 3 York Health Economics Consortium Ltd., Enterprise House, Innovation Way, University of York, York YO10 5NQ, UK; Julie.glanville@york.ac.uk; 4 York Trials Unit and NIHR RDS YH, Department of Health Sciences, Faculty of Science, ARRC Building, University of York, Heslington, York YO10 5DD, UK; catherine.hewitt@york.ac.uk

**Keywords:** systematic review, drug treatment, economic appraisal, offenders

## Abstract

*Background:* The numbers of incarcerated people suffering from drug dependence has steadily risen since the 1980s and only a small proportion of these receive appropriate treatment. A systematic review to evaluate the effectiveness and economic evidence of non-pharmacological interventions for drug using offenders was conducted. *Methods:* Cochrane Collaboration criteria were used to identify trials across 14 databases between 2004 and 2014. A series of meta-analyses and an economic appraisal were conducted. *Results*: 43 trials were identified showing to have limited effect in reducing re-arrests RR 0.97 (95% CI 0.89–1.07) and drug use RR 0.90 (95% CI 0.80–1.00) but were found to significantly reduce re-incarceration RR 0.70 (95% CI 0.57–0.85). Therapeutic community programs were found to significantly reduce the number of re-arrests RR 0.70 (95% CI 0.56–0.87). 10 papers contained economic information. One paper presented a cost-benefit analysis and two reported on the cost and cost effectiveness of the intervention. *Conclusions:* We suggest that therapeutic community interventions have some benefit in reducing subsequent re-arrest. We recommend that economic evaluations should form part of standard trial protocols.

## 1. Introduction

The number of incarcerated people suffering from drug dependence has steadily risen since the 1980s. Prevalence studies have produced a wide range of estimates for male (10% and 48%) and female (30% and 60%) prisoners [1,2,3,4,5,6]. Differences in the estimates, (according to Fazel and colleagues) are due to different measurement methods. Large numbers of offenders also suffer from substance misuse problems and have been consistently reported as a major contributing factor in the increasing population of women offenders [7,8]. The relationship between drugs and crime is also complex. The literature has discussed the issue of whether drug use leads people into criminal activity or whether those who use drugs are already predisposed to such activity. Nevertheless, the research evidence suggests that only a small proportion of these receive appropriate treatment and supervision [9]. In addition, the combination of drug use and offending behavior has a substantial economic impact on society and specifically on formal service resources [10].

Evidence about the effectiveness of different treatment options for drug-using offenders stem from previous systematic reviews and supervision models such as case management have been employed to smooth the transition process for individuals leaving prison to rehabilitation within the community [11,12]. Case Management in the U.S. has been applied in Treatment Accountability for Safer Communities (TASC) programs [13] and has shown initial effectiveness but without systematic evidence in support of the process.

Other forms of supervision include Intensive Supervision Programs (ISPs), combined with the use of vouchers, motivational interviewing (MI), and contingency management [14,15,16]. ISPs have been used as a type of sentencing order to extend the use of intermediate sanctions and available alternative options for imprisonment by imposing restrictive release conditions. ISPs also include elements of surveillance, monitoring, random testing, and multiple weekly contacts with a supervising officer. ISPs have increasingly been viewed as an alternative to relieve prison crowding and reducing risks to public safety. Assumptions about the effectiveness of ISPs on crime control involve comparisons of various types of sanctions and provide more control than routine supervision but less control than prison, and theoretically, offenders in ISPs are deterred from committing crimes because they are under surveillance. The additional use of voucher incentive schemes have been used to support prosocial, non-drug related behaviors to initiate new behaviors that reinforce positive consequences which help to sustain abstinence [14].

Other well established treatments for substance use in the U.S. and the UK include Therapeutic Communities (TCs). In the U.S., TCs combined with work release programs have been used since the 1950s to rehabilitate offenders. Previous meta-analyses and quasi-systematic reviews of incarcerated treatments (including TC evaluations) have shown modest effects in the reduction of recidivism and drug use [17,18,19].

Evaluations of adult and juvenile drug courts have been employed to enforce legal and moral sanctions. The court system acts as a diversionary scheme to avoid incarceration or return to prison for many offenders with drug use problems. Drug courts often involve the monitoring of drug abstinence using frequent urine testing and judicial monitoring. These mechanisms are used with a combination of collaborative processing and early identification to ensure the integration of drug treatment alongside the judicial system. Systematic review evidence evaluating the effectiveness of drug courts [20] identified three experimental trials of adult drug courts which demonstrated a modest (but non-significant) reduction in recidivism. One experimental juvenile court evaluation also showed a non-statistical significant result. The authors conclude that the evidence supporting the use of adult drug courts is tentative but points towards a subsequent reduction in offending and that further evaluations of juvenile drug courts are required to provide convincing evidence of their effectiveness. Drugs testing which often forms part of the drug court process is based on several theories or sets of assumptions including surveillance, early identification, prevention, and intervention. Alongside testing, voucher incentives schemes have been used to encourage abstinence from drug use with mixed effects [21,22,23,24]. 

Behavioral and psychological therapies including MI, multi-systemic therapy (MST), and cognitive behavioral therapy (CBT) have also been evaluated. Two previous systematic reviews of MI found that MI can lead to improved retention in treatment, but with mixed evidence for reducing substance use and offending behavior [25,26,27]. 

For cognitive behavioral treatment, large numbers of quasi-experimental and experimental studies have been conducted, although few have specifically focused on cognitive behavioral treatments for drug using offenders. Cognitive behavioral approaches are based on psychological theory and their application to support the rehabilitation of offenders has been extensively researched [28]. The theory emphasizes individual accountability and is used to enhance the recognition of thinking styles immediately preceding the criminal act. Treatment programs usually consist of a number of key elements including self-monitoring, identifying deficient thinking, and restructuring cognition [29,30,31]. Previous meta-analyses have excluded evaluations focusing on the needs of drug using offenders [30] but show a reducing in re-offending behavior generally.

Finally, a number of educational treatment programs containing elements of cognitive skills have been initiated through interactive online services. Interactive journaling uses learning strategies based on the Trans-theoretical Model of Change (TMC; [32]) and Motivational Enhancement Therapy (MET; [33]). Interactive journaling is designed to aid individuals to examine their feelings and cognitions surrounding maladaptive behaviors via interactive journaling booklets. The combination of emotional and cognitive expression used in interactive journaling has been shown to be more effective than cognitive processing alone in regard to behavior change [34]. Interactive journaling is a particularly appealing brief intervention for local jails because it requires minimal interaction by clinical personnel. The treatment has been shown to be effective in reducing the likelihood of engaging in serious forms of misconduct during incarceration among federal prison inmates, but has no previous systematic review evidence (e.g., [35]).

To our knowledge, there has been no previous systematic review investigating the effectiveness of treatment specifically for drug-using offenders across a number of different treatment options, and in some cases areas have been extensively researched (e.g., use of CBT) but studies have not focused specifically on populations of drug using offenders. As a consequence, we do not know whether such benefits transfer to this particular population. As this group of people forms a large proportion of the criminal justice system, it seems sensible to look at what specific treatment options might be effective in reducing subsequent offending and drug use behavior. Second, the present review goes beyond previous reviews by conducting a series of meta-analyses which shows evidence from Randomized Controlled Trials (RCTs) as opposed to quasi experimental studies. Other systematic reviews have taken the stance to include both quasi-experimental and experimental studies, thus generalizing the effectiveness of a particular intervention but potentially opening the possibility of compromise by study design. In principle, the RCT design eliminates the threat to internal validity providing there is a sufficiently large number of units assigned as the experimental and control conditions are equated [36]. As a result, the findings of this review may differ from previous reviews. To our knowledge, we are not aware of any review which takes a broad perspective investigating treatment for drug using offenders across a range of criminal justice settings and including multiple treatment options. Given the importance of relating economic cost to a reduction in drug use and related offending behavior, good quality economic evidence will help inform strategies which represent the best use of limited resource to reduce drug use and therefore improve associated outcomes [37]. The review also uses an economic protocol to evaluate the available resource information. According to Drummond studies containing information on the economics on the intervention are defined as full economic evaluation studies, partial economic evaluation studies, and single effectiveness studies. Full economic evaluations are the comparative analysis of alternative courses of action in terms of both costs (resource use) and consequences (outcomes, effects), [38].This differs from studies which focus solely on costs and resource use, or partial economic evaluations. Studies that use a full economic evaluation do not generally use a single research method; and aim to describe, measure and value all relevant alternative courses of action (e.g., intervention X versus comparator Y), their resource inputs and consequences, are referred to as a Cost-benefit analysis (CBA). Other evaluations which do not take into account all consequences include cost-effectiveness analysis (CEA) and cost-utility analysis (CUA). In this review, we use the Drummond Checklist to evaluate and document the availability of resource information within the studies.

The review has three primary research questions: (1) Do non-pharmacological treatments for drug-using offenders reduce criminal activity? (2) Do non-pharmacological treatments for drug-using offenders reduce drug use? (3) What economic resource, cost, and cost effectiveness information can be identified from these studies?

## 2. Methods

### 2.1. Search Strategy for Identification of Studies

For this review we searched 14 databases—CENTRAL (1980 to April 2014), MEDLINE (1966 to April 2014), EMBASE (1980 to April 2014), Psyc INFO (1978 to April 2014), Sci Search (Science Citation Index) (1974 to April 2014), Social Sci Search (Social Science Citation Index) (1972 to April 2014), NTIS (1964 to April 2014), Sociological Abstracts (1963 to April 2014), HMIC (to April 2014), PAIS (1972 to April 2014), Criminal Justice Abstracts (1968 to April 2014), LILACS (2004 to April 2014), Current Controlled Trials (December 2009)—from database inception up until April 2014. We developed individual search strategies for each database and made use of any controlled vocabulary—example search terms included: offender, criminal, inmate, substance use, prisoners, intervention, drug use, psychosocial, motivational, court, cognitive, relapse, and social skills. This included methodological search filters from the Inter-TASC Information Specialists’ Sub-Group (ISSG) Search Filter Resource site designed to identify Randomized Controlled Trials (RCTs). If filters were unavailable from this site, we substituted search terms based on existing versions (please contact the author for full details). In addition to the electronic databases, we searched relevant Internet sites (e.g., Home Office, National Institute of Drug Abuse (NIDA) and European Association of Libraries and Information Services on Alcohol and Other Drugs (ELISAD)) and we scrutinized the reference lists of all retrieved articles for further references. We undertook catalogue searches of relevant organizations and contacted experts for their knowledge of other published or unpublished studies relevant to the review. All references were placed in a bibliographic database for further scrutiny.

### 2.2. Criteria for Inclusion and Exclusion of Studies

#### 2.2.1. Selection Criteria

Studies included in the review were identified using a number of different criteria. These considered the study design (e.g., experimental or qualitative studies), the type of participants, and intervention description. We also pre-specified our outcome measures to avoid the possibility of any subsequent bias in choosing outcomes which might favor the effectiveness of our chosen interventions.

#### 2.2.2. Study Design

RCTs evaluating interventions to reduce, eliminate, or prevent relapse in drug using offenders were included. The comparison arm could contain any of the following: no treatment, minimal treatment, a waiting list, treatment as usual, or another treatment alternative. We categorised studies into any non-pharmacological intervention vs. waiting list control, treatment as usual, or vs. another treatment alternative.

#### 2.2.3. Selection of Participants

Drug-using offenders were included in the review regardless of gender, age, psychiatric history, or ethnicity. Offenders were defined as individuals who were involved in the Criminal Justice System (CJS). Offenders could reside in police custody, secure establishments (e.g., special hospitals, prisons or jails), or in the community (i.e., under the care of the probation, judicial court system or parole services). Drug use referred to individuals using occasional drugs or those who were considered drug dependent. 

#### 2.2.4. Intervention Type

The review included any Non-Pharmacological intervention; which was designed to reduce, eliminate or prevent relapse to drug use and/or criminal activity. As a result, a range of different types of interventions were included using (1) supervision models in the community and on release from prison, (e.g., case management); (2) therapeutic environments with and without work release programs; (3) drug courts and use of drugs testing (in combination and/or alongside supervision orders; (4) behavioral and psychologically-based therapies such as cognitive behavioral therapy, or MI; and (5) education-based interventions. 

#### 2.2.5. Outcomes Measures

We identified two primary outcomes: drug use and criminal activity. No restrictions were placed on drug use or drug dependence with all types of drug use considered including self-report and biological measures of drug use such as hair or urine analysis. Self-report and official custody records of criminal activity were included in the analyses. Measurements of criminal activity included arrest for any offence, drug offences, re-incarceration, convictions, charges, and recidivism—recidivism specifically referred to individuals who engaged with any form of criminal activity following release from an incarcerated setting—alongside our secondary outcome reporting on resource use, cost, and cost-effectiveness. Papers reporting on both alcohol and drug use outcomes were included in the review when these outcomes were reported separately.

#### 2.2.6. Excluded Studies

Offenders with alcohol problems were excluded from the review along with any specific treatment for alcohol use. For example, specific courts for Driving Whilst Intoxicated (DWI) or motivational interview techniques aimed at offenders with alcohol addictions were excluded. Additionally, boot camp interventions for drug using offenders were excluded due to their lack of effectiveness in a previous systematic review [39].

#### 2.2.7. Screening and Coding Process

Five independent authors inspected the titles and abstracts for potential inclusion in the review. Of those identified, the full articles were obtained for each paper and assessed for full inclusion. In the case of discordance, an independent author arbitrated. The translations of two articles written in Spanish were undertaken by a single reviewer. Where missing data occurred in the original publication, the study author was contacted via email. We developed a standardized protocol for the purpose of data extraction which matched that required by the Cochrane Collaboration. Two pairs of reviewers extracted data and then subsequently agreed on the final data extraction. The coding items included information about the study sample, intervention, and control groups and the key results for our outcome measures.

#### 2.2.8. Quality Assessment

An independent quality assessment was conducted using risk of bias assessment criteria recommended in the *Cochrane Handbook for Systematic Reviews of Interventions* [39]. The recommended approach for assessing risk of bias in studies included in a Cochrane Review involves the use of a two-part tool that addresses six specific domains, namely: sequence generation and allocation concealment (selection bias), blinding of participants and providers (performance bias), blinding of outcome assessor (detection bias), incomplete outcome data (attrition bias), selective outcome reporting (reporting bias), and other sources of bias. This provides a rating of either low medium or high risk of bias.

#### 2.2.9. Statistical Methods

**Figure 1:**
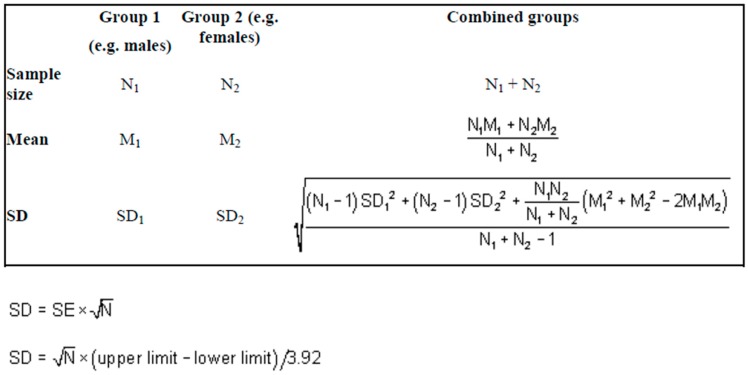
Statistical treatment.

The Revman software package (Review Manager 2014) was used to perform a series of meta-analyses for continuous (using Mean Difference: MD for measures on different scales and 95% Confidence Intervals) and dichotomous outcome measures (using Relative Risk: RR and 95% Confidence Intervals). A random effects model was used to account for participants coming from different underlying populations. We used the transformations as laid down by the Cochrane Handbook for continuous outcomes (see Section 7.7.3) and where appropriate we combined intervention and control groups to create a single pair wise comparison. Where this was not appropriate, we selected one treatment arm and excluded the others. For conversions of Standard Error into Standard Deviations and the calculation of Standard Deviations calculated from 95% CIs we used the standard equations set out in Figure 1. 

For outcomes of criminal activity enough data allowed us to present outcomes of re-arrest and re-incarceration separately. We favored effect sizes that were given for criminal activity data of (1) arrest generally, as opposed to a specific offence; and (2) re incarceration to jail, prison, or other secure setting. A number of common dependencies were created by multiple measures of both outcomes (e.g., arrest and parole violation) and follow up time periods (e.g., 12, 18 months). All trials were checked to ensure that multiple studies reporting the same evaluation did not contribute towards multiple estimates of program effectiveness. We chose to report on the longest outcome measure to provide the most conservative estimates of effect. This meant that studies with multiple follow-ups were represented by only one study in the meta-analysis. We considered study heterogeneity using the Cochrane handbook guidelines (Section 9.5.2) where *I*^2^ were considered using the following rough guidelines: 0%–40%: might not be important; 30%–60%: may represent moderate heterogeneity, 50%–90%: may represent substantial heterogeneity, 75%–100%: considerable heterogeneity.

#### 2.2.10. Economic Appraisal

Any available economic or resource information was assessed using the Drummond Checklist shown in Table 1 [38]. This criterion was applied by an economist to indicate the cost of the intervention and the consequences of intervention on resources and costs relevant to various public sectors. These included healthcare, criminality, labour force participation, or other public goods. Evaluations according to Drummond need to be comparative as an intervention can only be labelled relative to a benchmark or alternative. Evaluations that are not comparative and do not consider both costs and consequences, and/or a comparator is classified as a partial evaluation (e.g., 1A, 1B, 2). A cost effectiveness or cost study is described if alternatives are compared (e.g., 3A, 3B). However, if only the costs or benefits are described the evaluation is still considered partial evaluation but would be comparative across one-dimension. A study evaluating all aspects of the economic dimensions and a comparative would be considered a full economic costing (e.g., 4).

## 3. Results

### 3.1. Search Findings

**Figure 2:**
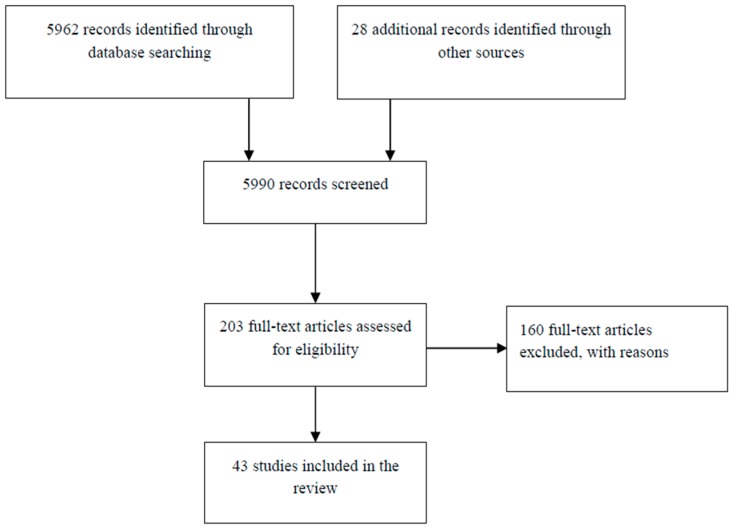
PRISMA Flow Diagram.

The updated searches spanned from database inception until April 2014. This identified a total of 5990 records. Of these 5787 were excluded on title and abstract screening. We acquired a total of 203 full text papers for assessment and following a full screen excluded 160 papers. The 43 trials produced 55 publications and represented 14,019 participants from research published between 1992 and 2014 (see Figure 2).

39 of the 43 trials were conducted in the U.S. The other four studies (producing five publications) were conducted in Sweden [40], China [41], Spain [42,43], and Australia [44]. Six trials did not specify the sample gender [15,40,45,46,47,48], six trials contained female only samples [34,35,44,49,50,51,52], nine trials (and 13 publications) contained only male adult offenders [53,54,55,56,57,58,59,60,61,62,63,64,65]; and 22 trials (and 25 publications) contained male and female adult offenders [13,16,41,44,66,67,68,69,70,71,72,73,74,75,76,77,78,79,80,81,82,83,84,85,86,87,88,89,90]. Eight comparisons considered young and/or juvenile offenders [16,49,56,57,66,67,83,91,92]. Seven comparisons contained majority black ethnic origin participants [47,52,60,68,71,72,73,78,85]. The studies were divided by setting into community (*n* = 18), prison and secure establishment (*n* = 13) and court based studies (*n* = 12).

Five trials represented data from multiple follow up publications [16,49,53,54,61,62,71,72,73,92]. The Gottfredson studies published data at 24 and 36 months and at five-year follow-up to the primary study [63,64]. A secondary analysis presented 12 month crime data [61]. The Sacks studies published data on two separate studies reporting 6 and 12 month outcomes and a secondary analysis of the data [49,53,54,92].

### 3.2. Overview of Studies

#### 3.2.1. Meta Analyses 

The meta-analyses divided studies into two main outcome measures: criminal activity (including self-report and official records of arrest and re-incarceration), and drug outcomes (biological and self-report). Finally, we considered the impact of specific types of interventions on criminal activity and drug use. Our interventions fell into five different groups: supervision in the community, drug and mental health courts, therapeutic communities, drug testing, and psychological therapies.

#### 3.2.2. Does Any Type of Non-Pharmacological Treatment for Drug Using Offenders Reduce Criminal Activity?

**Figure 3:**
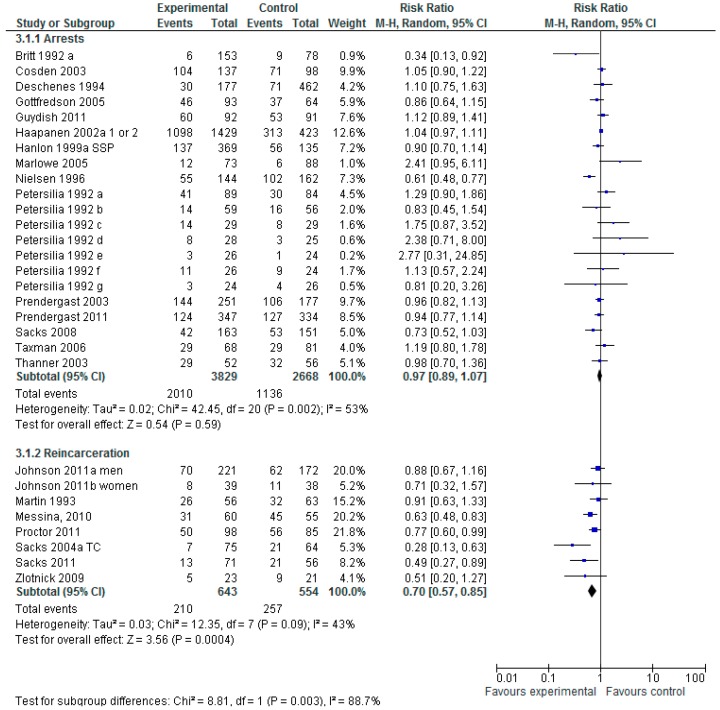
Does any type of Non-Pharmacological intervention reduce subsequent criminal activity?

Figure 3 shows the analysis of a random effect model to evaluation the impact on subsequent arrests (6497 participants) and re-incarceration (1197 participants). For measures of arrest a total of 21 studies are combined, the Risk Ratio and 95% CI show that these interventions overall do not statistically significant reduction in subsequent arrests RR 0.97 (95% CI 0.89–1.07), but where moderate levels of heterogeneity exist. For measures of re-incarceration, a total of eight studies are combined, the Risk Ratio and 95% CI show that these interventions do statistically significantly reduce subsequent re-incarceration RR 0.70 (95% CI 0.57–0.85), but where potential substantial levels of heterogeneity exist.

#### 3.2.3. Does Any Non-Pharmacological Treatment for Drug Using Offenders Reduce Drug Use?

**Figure 4:**
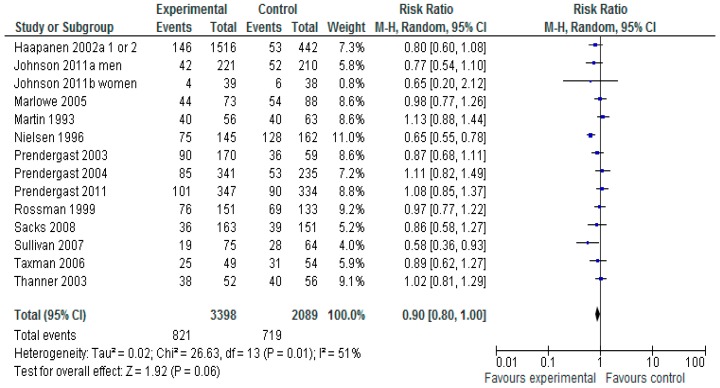
Does any type of Non-Pharmacological intervention reduce subsequent drug use?

Figure 4 shows the analysis of a random effect model to evaluation the impact on subsequent drug use (5487 participants). For measures of self-report and official dichotomous drug use a total of 14 studies are combined, the Risk Ratio and 95% CI show that these interventions overall do show a near-statistical significant reduction in subsequent drug use RR 0.9 (95% CI 0.80–1.00), but where moderate levels of heterogeneity exist.

#### 3.2.4. Does Intervention Type Have an Impact on Subsequent Criminal Activity?

**Figure 5:**
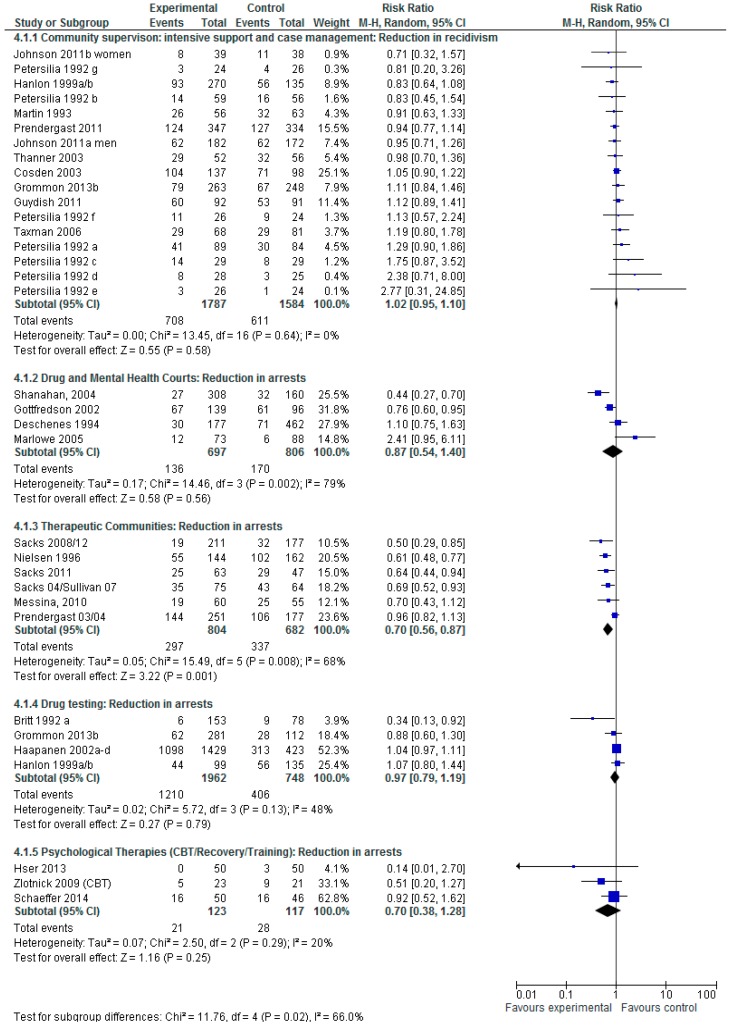
Does Intervention type impact on subsequent criminal activity?

Figure 5 shows the analysis of a random effect model to evaluate the impact on subsequent criminal activity by intervention type. For community supervision (including intensive support and Case Management) 17 studies are combined, the Risk Ratio is 1.02 and 95% CI (0.95–1.10) show that these interventions do not statistically significantly reduce subsequent recidivism, where heterogeneity is likely for these studies not to be important. For drug and mental health courts four studies are combined, the Risk Ratio is 0.87 and 95% CI (0.54–1.40) show that these interventions together do not statistically significantly reduce subsequent re-arrests, where heterogeneity between the studies is likely to be considerable. Studies evaluating therapeutic communities with and without work release programs combined six studies, the Risk Ratio is 0.70 and 95% CI (0.56–0.87) showing that these programs do produce a subsequent reduction in re-arrests, but where heterogeneity is likely to be substantial. Four studies evaluating drug testing showed no significant reduction in re-arrests RR is 0.97 and 95% CI is (0.79–1.19), but where heterogeneity is likely to be moderate. Three studies using psychological therapies including cognitive behavioral therapy and recovery training showed no subsequent reduction in re-arrests RR 0.70 and 95% CI is (0.38–1.28) but where heterogeneity is likely to be substantial.

#### 3.2.5. Quality Assessment

**Random sequence:** Although all the included studies were randomized trials, over two-thirds of them (*n* = 27) provided an inadequate description and were rated as unclear by the reviewers. In a further eight studies a good description of the random sequence was provided resulting in a rating of low risk of bias [48,57,64,67,70,78,85,92]. The final five studies were rated at high risk of bias presented random sequence methods which caused concern [40,45,52,65,68].

**Allocation concealment:** In two-thirds of all studies (*n* = 31), the majority of descriptions about the method of allocation was unclear. Three studies provided adequate descriptions and were therefore rated at low risk of bias [14,52,63]. The remaining six studies were classified as high risk of bias [40,45,67,74,85,86].

**Blinding performance:** Blinding in some trial participants was not feasible due to the study design, however some studies did attempt to blind outcome assessors where possible. In the majority of studies, blinding was rated as unclear by the reviewers (*n* = 25). In a further seven studies, risk of bias was considered low [40,52,56,57,68,78,92]. The remaining eight studies were rated at high risk of bias from a lack of blinding [14,45,50,63,64,74,85,86].

**Incomplete outcome:** Unclear reporting of incomplete outcome measures was apparent in the majority of studies (*n* = 32). In the remaining eight studies, five were rated at low risk of bias [50,52,78,83,91] and three were rated at high risk of bias [60,69,84].

**Selective reporting:** The majority of papers were rated as unclear (*n* = 33), not clearly specifying primary and secondary outcome measures. In six studies, pre-specified outcomes were presented and were rated as low risk of bias [14,67,75,78,83,86] and two studies presented a high risk of bias [74,92].

**Other bias:** In many instances (*n* = 25), studies were rated as unclear on other bias. Two studies were rated at low risk of bias [65,83] and the remaining 12 studies were rated at high risk of bias [16,48,50,60,69,73,75,76,78,84,92].

## 4. Economic Appraisal

The economic appraisal using the Drummond criteria identified 10 studies reporting some cost information. Table 2 shows the results of the type of resource costs described for each paper. Five of the 10 papers reported costs associated with the intervention, eight papers reported information relating to healthcare costs, all 10 reported on costs associated with criminality, four reported on productivity costs, and two reported on other public goods. Only 1 of the 10 papers reported on all four areas [48].

In terms of the overall Drummond classification, three studies were reported as “4” and represented a full economic appraisal. These represented one cost-benefit analysis [48] and two cost effectiveness analyses [93,94]. The Rossman study evaluated the “Impact of the Opportunity to Succeed (OPTS) Aftercare” program costs (USD $1,810 over 1–2 years of the program) as well as benefits (valued in monetary terms) of the program to usual services (USD $105,339 compared USD $108,632). The two cost effectiveness papers report on the costs but not benefits of the program outcomes for an Adult Drug Court (ADC) vs. an alternative to jail for criminal offenders addicted to illicit drugs [94] and a community based MST program [93]. The ADC was reported as cost effective in delaying the time to first offence and in reducing the frequency of offending for those outcome measures selected. The authors noted a number of limitations with the data which meant that they were unable to investigate the relative effectiveness of different treatment modalities and the estimated costs for the sentencing process were conservative and were based on a small group of participants based in a local court [94]. The MST program for young offenders was compared to usual care. The study calculated the incremental costs of MST and observes reductions in days of incarceration, hospitalization, and residential treatment at approximately one-year post-referral. Individual outcomes for the program were included in the economic analysis and the results of an additional USD $877 cost per young person for the therapy were estimated.

The remaining seven studies represent different partial evaluations (i.e., cost and/or benefits which are not incremental relative to the control). Two compare the effectiveness of interventions providing the absolute cost of the intervention only [49,92]. Research in [69] combined reported on the effectiveness of a contingency management programme attached to a drug court system. The paper contains some detail of the payments made and the costs of such a contingency programme between 2002 and 2004. The two different incentive schemes were implemented in the treatment group and are described to have average costs of $122.83 (SD 133.89) for an escalating reward scheme and $150.39 (SD 109.65) for the non-escalating reward scheme. No description of resource required and/or cost of drug court as usual (the control group) is provided. Furthermore, these monetary values are for “gift certificates earned” in the control group and this may not capture the full direct cost of implementing either version of the contingency management programmes. Sacks (2004, [53]) evaluated a modified TC for male adult offenders with a serious mental disorder and substance use. The study contains information about the cost of providing a therapeutic community intervention. For this intervention, the additional marginal costs on top of the specific incarceration costs were estimated at USD $7.37 per day. However, it is unclear from the study how this “marginal” cost over incarceration was obtained (via an incremental comparison versus control or a unit cost estimated from previous economic research).

Four further partial evaluations comparing the effectiveness of interventions providing resource implications or costs as outcomes [15,83,84,91]. Henggeler 1999 (associated with Schoenwald 1996, [83,93]) compares the effectiveness and transportability of MST in juvenile offenders meeting DSM-III-R criteria for substance abuse. As an analysis of effectiveness the study reports resource implications of MST on use of drugs, and the total days out-of-home, none of which are associated with their unit costs within this paper. McCollister (2009, [84]) reports on the differential costs of criminal activity in juvenile drug court participation using the Henggeler (2006, [91]) study as an example. The economic study estimates the cost of criminal activity for nine specific crimes at baseline, 4, and 12 months. No information on difference in the resources required and/or costs of “juvenile drug court” were given to indicate variation in inputs required by the system. The paper reports on a number of methodological challenges suggesting that it may be more difficult to economically quantify frequency and type of criminal activity for adolescents than for adults. Furthermore, the paper proposes guidelines for future economic evaluations of adolescent substance abuse and crime prevention programs. Petersilia (1992, [15]) suggests that there is an additional cost of USD $3,000 per annum (1992 prices) for intensive probation supervision. On cost comparison, the costs per day are lower or comparable to the additional costs per day of the therapeutic community in prison. An evaluation of a case management release program referred to as “opportunities to succeed scheme” estimated the program service provision costs (excluding administration costs). The final study by Chandler (2006, [95]) examines the use of high fidelity Integrated Dual Disorders Treatment program in jail recidivists with serious mental illness and substance use disorder. This study compares of arrests, convictions, jail days, and mental health costs. Mental health costs were higher at baseline in the intervention group than control (USD $3,556 vs. $1,490) and over the period of the study (USD $9,176 vs. $6,318) indicating an overall incremental increase of USD $792. The study shows between differences in criminality however unit costs of crime were not applied.

## 5. Discussion

Overall, the findings from our review suggest that non-pharmacological interventions for drug-using offenders have little impact on reducing self-report drug use and subsequent re-arrests when combined together. However, some significant reductions are shown in relation to subsequent re-incarceration. Reasons for differential results between outcome measures may be due to the way in which different outcomes are measured. For example, re-arrest is relatively immediate as opposed to re-incarceration which can take many months to occur and record on the criminal justice system. A reduction in re-incarceration may also reflect that individuals are committing fewer crimes of a serious nature and instead may be re-arrested for lesser charges. Evaluations of the impact of individual interventions models revealed that therapeutic community interventions were shown to significantly reduce subsequent re-arrest. This finding supports other systematic reviews in the field [16,17,18]. Not covered in this review, but noted elsewhere, is the importance of TC’s combined with aftercare and work release programs which appear to have greatest impact. Most recent findings of a modified TC show just 9% of offenders were re-imprisoned compared to 33% after usual procedures. The growing numbers of evaluations that show evidence of treatment effectiveness for prison TC treatment followed by aftercare have important policy and practice implications [49]. Specifically, the work suggests that support of such participation and continued engagement in aftercare services play an important role in continued rehabilitation of offenders with substance use problems. The survival analysis conducted by [66,67] suggested that engagement in the community was most important in the first two months following release when individuals were most vulnerable. From a practical point, this suggests that parolees enrolled into community programs as a condition for parole would encourage liaison between the provision of drug use services in prison and the community. Such findings are supported by an Independent Commission report in the UK which stressed that post-prison continuing care is required to maintain the rehabilitation of such offenders (Home Affairs Committee, 2012, [96]). Nevertheless, one of the major drawbacks of prison TCs is that these are usually reserved for prisoners with at least 12 months left to serve, excluding many offenders with substance use problems.

None of the other four interventions (community supervision, drug and mental health courts, drug testing, and psychological and behavioral therapies) were shown to reduce re-arrests. These findings broadly concur with other systematic reviews in the field with the exception of the literature relating to the effectiveness of cognitive behavioral therapies [30,31,32,33]. Previous reviews in this area have supported the use of cognitive behavioral therapies for offender populations but have excluded drug using offenders specifically from their study evaluations. Our findings (whilst contrary to previous research) represent three studies of psychological and behavioral therapies containing in total 240 persons evaluating outcomes of arrest. As such, the findings should be interpreted with caution.

Appraisal of economic evidence currently identified studies containing cost information relevant to drug use interventions in offenders. Most commonly, studies examined whether intervention(s) may alter criminality as outcomes and describe the consequences for resources and public sector costs. However, there is substantial variation in the framework adopted for evaluations and only a limited number meet the basic criteria for a full economic evaluation. Reviews of economic evaluations of criminal justice interventions found that the sector is less amiable to fully adapt health economics methods than, say, social care. Shemilt et al. (2010, [97]) identify three main reason for this: (1) social well-being (including that of victims) is hard to measure; (2) cost-effectiveness analysis tends to adopt intermediate measures of crime without seeking links to metric analogous to well-being (i.e., QALY) leading to “tax-payer” perspective and hence no clear budget; and (3) difficulty in estimating cost of cost and have been explored by the European Commission. The authors point to a lack of a centralized strategy in incorporate economic evidence into criminal justice policy-making.

Taking their limitations into account, there is evidence to suggest interventions have consequences on future criminality [48,94] or healthcare demand [93] and overall may offset the cost of intervening. However, authors indicate limitation in their studies’ ability to fully capturing relative effective or associated costs [94] and methodological challenges in monitoring and qualifying consequences indicate the need for better and agreed guidelines [84].

The majority of studies identified as “containing costs” fall short of meeting criteria for an economics of the intervention. Such studies can be broadly divided into two groups: either the study does not fully quantify direct cost of intervention versus cost of in control group (i.e., costs of the inputs of treatment); or it does not consider the consequences of treatment to health, criminality, or wider society (i.e., costs of the outputs from treatment). However, studies not meeting criteria commonly provide detailed comparison of resource consequences (particularly of crime rates) and may have sufficient information to apply associated unit costs (e.g., cost of specific crimes) to develop a full economic evaluation. In general, there is less detail on resources or cost of the input in providing interventions and therefore there is limited basis to include this to estimate total costs.

The three full economic evaluations identified illustrate minimum standards for future economic evaluation conducted alongside single empirical studies. They indicate that intervention costs (inputs) as well as cost consequence (outputs) of the alternative need to be examined, and those studies need to compare two or more alternative to routine care. With regard to informing policy, the appraisal identified resource information on healthcare, criminality, labour force participation, or other public goods, all of which indicates that a variety of public sector and wider societal perspectives may be adopted by various decision-makers. Future studies aiming to inform policy on non-pharmacological interventions for drug use in offenders, should primarily consider the appropriate perspective and, given the inter-sectoral nature of required financial inputs, consensus exercise should enlist decision-makers in healthcare and criminal justice to agree and contract required information to implement future policies.

## 6. Limitations

In summary, the extent to which we can say interventions (with the exception of therapeutic community interventions) for drug using offenders reduces subsequent drug use and criminality is limited and the current evidence raises the possibility that overall treatment for drug using offenders may not be effective in reducing subsequent drug use or criminal activity; or that any effect that is produced is small and in some cases shows great heterogeneity between studies. Despite the relatively large number of trials, almost all were conducted within the U.S., thus limiting the applicability and external generalisation of the findings to other countries with different judicial systems. Nevertheless, the growing body of evidence demonstrates the significant financial support for such evaluations and a commitment to an investment in the delivery and implementation of interventions aimed at helping drug using offenders reduce subsequent drug use and criminal activity.

The quality assessment process highlighted a number of limitations within each of the studies—these include small sample sizes, loss to follow-up, and selection bias. A number of trials were defined as pilot studies with relatively small sample sizes [85], and with large amounts (30%) of attrition and loss to follow-up making it difficult to derive definitive conclusions , (e.g., [40,74]). The Stein (2011, [57]) study was noted as relatively underpowered however it does represent one of the first randomized controlled trials in the juvenile correctional facility. The replication of such study findings are required to ensure the generalization and external validity of the findings. 

Selection bias was a concern in the Wexler studies whereby participants were randomly assigned to the prison TC and regular prison conditions but not to aftercare. The authors note that possible differences in personal motivation may account for some of the positive outcomes associated with participants continued support for aftercare services. Subsequently, these participants were noted as having the highest “readiness scores” which suggests that motivation creates an important consideration on client selection Wexler (1999b, [64]). Selection bias was noted as a concern in the Prendergast (2003/4, [61,62]) study whereby prison dropouts, prison completers, aftercare dropouts, and aftercare completers were the results of self-selection, due to the voluntary nature of participation in the aftercare phase of the study. This may result in the most highly motivated or successful individuals completing treatment and the results may be due partially to the client characteristics rather than the treatment alone.

With these concerns in mind, the studies showed an overall degree of statistical variation and caution in the interpretation of the results is required. We did not explore other possibilities such as differences between gender, age, and length of follow up in our analysis and this will be something to address in the subsequent update of this review. We have some, but not adequate information on the costs and costs effectiveness of these interventions. It would be helpful for researchers to include an economic evaluation as part of the protocol. Program developers and evaluators should integrate and work closely with economists during the early planning and implementation stages to ensure that the best measures and data are collect for economic evaluation objectives. For example, the Drug Abuse Treatment Cost Analysis Program (DATCAP: http//www.datcap.com) provides estimates of total program costs, weekly cost per client, and the average cost per treatment episode. Such individual level cost data can then be directly compared to economic outcomes to estimate the costs and benefits of a program or intervention.

## 7. Conclusions

Despite the relatively large number of trials included in this review, the differential results displayed with some outcomes (but not others) suggest that a standardized list of accepted outcome measures is required. Qualitative research and translational work in this area of research may help to unlock some of the mechanisms underlying the principles of some of these interventions (e.g., therapeutic communities and multi-systemic therapy). These might include such relevant factors including inmate engagement, prior treatment experiences, and post intervention treatment experiences. Research including the long-term follow-up of offenders may help to answer the longevity of impact and it would be interesting to explore further whether these interventions can be used to target specific re-offending behavior.

## Figures and Tables

**Table 1:** Classification scheme for economic evaluations (Drummond 2005).

*Are both Costs (Inputs) and Consequences (Outputs) of the Alternative Examined?*				
***Are two or more alternatives compared?***	**No**	**No**		**Yes**
		Examine consequences only	Examine only costs	
		1B PARTIAL EVALUATION 1B		2 PARTIAL EVALUATION
		Outcome Description	Cost description	Cost-outcome description
	**Yes**	3A PARTIAL EVALUATION 3B		4 FULL ECONOMIC EVALUATION
		Efficacy effectiveness evaluation (e.g., RCT)	Cost analysis	Cost effectiveness analysis Cost Utility analysis Cost benefit analysis

**Table 2:** Available economic information (resource use and/or cost) and evaluation type according to Drummond Classification Scheme (see Table 1).

Author (Year)	Sample Description	Intervention Summary	Does the Study Describe Resources Use AND/OR Costs for:					Drummond Score
			Interventions	Healthcare	Criminality	Productivity	Other Public Goods	
Chandler & Spicer (2006)	Jail recidivists with serious mental illness and substance use disorder	Dual Disorder Treatment program	-	√	√	-	-	3B
Henggeler (1999)	Young offenders	Community based MST [2]	-	√	√	-	√	3A
Henggeler (2006)	Young offenders	Family and Drug Court with Community Services including MST and enhanced contingency management	-	√	√	-	-	3A
Marlowe (2008)	Male adult offenders with no more than two previous convictions and in need of treatment for drug dependence	Drug court and contingency management programme.	√	√	√	-	-	3A
McCollister (2007)	Juveniles offenders meeting the diagnostic criteria for substance abuse	Drug court combined with a number of different therapies.	-	-	√	√	-	3B
Petersilia (1992)	Male adult offenders sentenced to community-based supervision	Intensive probation supervision.	-	-	√	√	-	3A
Rossman (1999)	Male adults referred to a community-based program.	Opportunity to Succeed Scheme.	√	√	√	√	√	4
Sacks (2004)	Male adult offenders with a serious mental disorder and substance use	Therapeutic Community [1]	√	√	√	√	-	3A
Schoenwald (1996)	Young offenders	Community based MST [2]	√	√	√	-	-	4
Shanahan (2004)	Male and female offenders referred to an adult drug court	Drug court	√	√	√	-	-	4
